# DADOS-Survey: an open-source application for CHERRIES-compliant Web surveys

**DOI:** 10.1186/1472-6947-6-34

**Published:** 2006-09-15

**Authors:** Anand Shah, Danny O Jacobs, Henrique Martins, Matthew Harker, Andreia Menezes, Mariana McCready, Ricardo Pietrobon

**Affiliations:** 1 School of Medicine, University of Pennsylvania, 3450 Hamilton Walk, Philadelphia, PA, 19104, USA; 2 Department of Surgery, Duke University Medical Center, Box 3704, Durham, NC, 27710, USA; 3 Center for Excellence in Surgical Outcomes, Division of Orthopaedic Surgery, Duke University Medical Center, Box 3094, Durham, NC, 27710, USA

## Abstract

**Background:**

The Internet has been increasingly utilized in biomedical research. From online searching for literature to data sharing, the Internet has emerged as a primary means of research for many physicians and scientists. As a result, Web-based surveys have been employed as an alternative to traditional, paper-based surveys. We describe DADOS-Survey, an open-source Web-survey application developed at our institution that, to the best of our knowledge, is the first to be compliant with the Checklist for Reporting Results of Internet E-Surveys (CHERRIES). DADOS-Survey was designed with usability as a priority, allowing investigators to design and execute their own studies with minimal technical difficulties in doing so.

**Results:**

To date, DADOS-Survey has been successfully implemented in five Institutional Review Board-approved studies conducted by various departments within our academic center. Each of these studies employed a Web-survey design as their primary methodology. Our initial experience indicates that DADOS-Survey has been used with relative ease by each of the investigators and survey recipients. This has been further demonstrated through formal and field usability testing, during which time suggestions for improvement were incorporated into the software design.

**Conclusion:**

DADOS-Survey has the potential to have an important role in the future direction of Web-survey administration in biomedical research. This CHERRIES-compliant application is tailored to the emerging requirements of quality data collection in medicine.

## Background

The Internet has been increasingly utilized in biomedical research. From online searching for literature to data sharing, the Internet has emerged as a primary means of research for many physicians and scientists. More recently, Web-based surveys have been employed as an alternative to traditional, paper-based surveys. Several studies have demonstrated that Web-surveys have comparable and, in many instances, facilitate higher-quality results than traditional surveys [1-4]. Notably, Web-based surveys have several advantages: speed of data collection, automated data entry, and less potential for human errors, to name a few.

However, the use of Web-based surveys in the biomedical sciences presents several inherent challenges. From issues regarding anonymity of survey participants [5], generality and validity of the data, and multiple responses from a single participant, Web-surveys present challenges for researchers who choose to use them [6]. Recently, the Checklist for Reporting Results of Internet E-Surveys (CHERRIES) was formulated to address many of these concerns [7]. Similar to the QUORUM guidelines for systematic reviews and CONSORT statement for randomized trials, the CHERRIES statement is the first checklist for ensuring the quality of biomedical research that utilizes Web-survey methodology. The CHERRIES statement has been described in detail elsewhere [7]. In short, CHERRIES mandates that results from Web-surveys are reported with the following issues adequately addressed: survey design, Institutional Review Board (IRB) approval and informed consent process, development and pre-testing, recruitment process and description of the survey participant sample, survey administration, response rates, prevention of multiple entries from the same individual, and data analysis.

In this article, we describe DADOS-Survey, the first-ever open-source Web-survey software application to, the best of our knowledge, fully integrate the CHERRIES guidelines into its design. DADOS-Survey provides Web-survey administrators with the necessary means to collect and report data that is compliant with CHERRIES. Although multiple Web systems exist for collection of survey data using customizable forms (Survey Solutions Express, Inquisite, SurveyWriter, SelectSurveyASP, Vista, all last accessed February 2006), to our knowledge, no system has been designed to readily ensure CHERRIES compliance. We believe that DADOS-Survey addresses the specific concerns regarding the use of Web-surveys in biomedical research. We outline below the design objectives, software architecture, implementation, usability, and future directions for DADOS-Survey.

## Implementation

### Design Objectives

DADOS-Survey was designed with usability as a priority, allowing investigators to design and execute their own studies with minimal technical difficulties. Philosophies such as User-Centered Design [8], which place the main focus of the website on the user, and Web 2.0 were implemented. In short, our general goals are summarized in Table 1.

**Table 1:** Design Objectives for DADOS-Survey

Usefulness	Ensuring that software functions match the user's needs, while maintaining compliance with CHERRIES guidelines
Visibility	Users should be readily able to locate and understand which functions are available
Navigation	Users should be able to easily navigate through the software website
Feedback	Users should be able to provide rapid feedback to website administrators for constant improvements
Simplicity	The more simple the application, the greater its utilization

Within the context of these goals, we outlined several specific objectives before designing DADOS-Survey:

1. The program should be fully compliant with the CHERRIES Web-survey guidelines.

2. Non-anonymous surveys, by definition, contain protected health information (PHI) as specified by the Health Insurance Portability and Accountability Act (HIPAA), and thus require informed consent. The user should therefore have the ability to provide informed consent for non-anonymous surveys.

3. The program should be able to deliver anonymous surveys, not containing PHI, and therefore not in need of informed consent.

4. The program should have a mechanism to prevent entry of non-invited respondents for anonymous and non-anonymous surveys.

5. The user can deliver personalized e-mails to potential respondents, including the name of the principal investigator as the sender. Potential replies to e-mails should be captured and stored by the server.

6. The program should be able to record the exact date and time when each respondent answered the survey.

7. The Web-survey should be able to randomize the item sequence of a survey form. Item randomization avoids framing of item responses by precedent items.

8. The user should be able to determine the number of items per page, thus avoiding long pages or different pages with an unequal number of items.

9. Survey participants must perform a "completeness review" on the last page of the survey, giving respondents the opportunity to review and change their responses.

10. For non-anonymous surveys, respondents should be tracked through their login and password. Individuals who have already responded should be prevented from responding again.

11. The application should be able to count survey responders when they arrive at the first page of the survey, e-sign the informed consent, and submit the survey, all while determining the time between each of these activities. This information will be used to validate survey results and facilitate sensitivity analyses.

12. The application should be able to include pictures and flash movies.

13. The application should be able to collect private information data in a secure (HTTPS) environment in compliance with HIPAA regulations

### Software architecture

**Figure 1:**
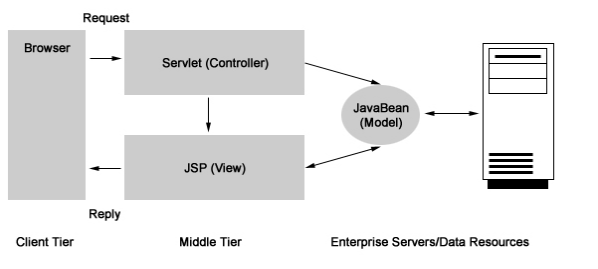
Model-View-Controller (MVC) design paradigm. [Adapted from Pietrobon R, Shah A, Kuo P, Harker M, McCready M, Butler C, Martins H, Moorman CT, Jacobs DO: Duke Surgery Research Central: an open-source Web application for the improvement of compliance with research regulation. *BMC Med Inform Decis Mak *2006, 6(1): 32]

The software architecture for DADOS-Survey is modeled after other software applications designed by our group [9]. In short, the software was developed using Java (Sun Microsystems Inc, Santa Clara, CA) as the programming language, Model-View-Controller (MVC2) as the design model, and because of the need for achieving functionality for feedback and system growth, the evolutionary prototyping was used. The idea behind the MVC design model is that an application consists of three components: a Model, Views of the Model, and Controllers. The Model is the part of the application that contains the actual application logic. The Model performs the database access, computes numbers, and manipulates data structures. The View and Controller represent the user interface of the application. The user interface is conceptually split into input and output components. The Controller is an input component that supplies information to the Model. The View is an output component that displays information from the Model. The View typically communicates with the Model by registering itself as a callback and responding to events generated by the Model [see Figure 1, adapted from Pietrobon R, Shah A, Kuo P, Harker M, McCready M, Butler C, Martins H, Moorman CT, Jacobs DO: Duke Surgery Research Central: an open-source Web application for the improvement of compliance with research regulation. *BMC Med Inform Decis Mak *2006, 6(1): 32].

For installation, Java >= 1.4.2 is required for APACHE Tomcat 5.X to run and as backend, MySQL 5.0.

### Navigation

**Figure 2:**
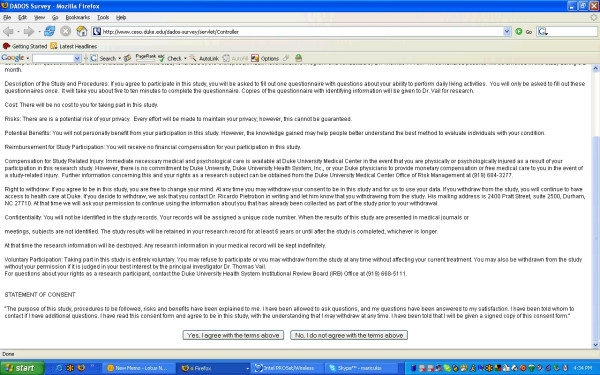
IRB consent for Web-survey participants using DADOS-Survey.

Each principal investigator (PI) is given access to the DADOS-Survey site and is subsequently able to design and publish a custom Web-survey for study purposes. Only the PI of the study has access to the project on the DADOS-Survey system. Once authenticated into the software program, the PI is able to input their surveys, IRB consent [see Figure 2], e-mail address list, and the body text for the e-mail that is sent out with each survey. The latter allows the PI to briefly introduce the study and its aims in an e-mail, at the end of which a URL allows the recipient to access the Web-survey. Depending on the study design, investigators are able to design an IRB consent form [see Figure 2], which survey recipients must review prior to progressing further into the survey.

Upon login, the user finds a list of the surveys that they are currently administering, or they have the option to create a new survey. Once an existing survey is selected or a new survey initiated, the investigator can modify the contents within it by using the toolbar across the top of the page. Each of the options in the toolbar is explained in detail in Table 2.

**Table 2:** Explanation of Survey Management Toolbar Options

Modify Study	Modify the Web-survey form
Import e-mail sheet	Import a Microsoft Excel list of e-mail addresses for survey recipients (a template is provided for users to download also)
Define e-mail	Create the recipient e-mail that is sent with each survey URL. This e-mail contains an automatically-generated, customized greeting based on the e-mail list, while indicating the PI as sender of the e-mail
Data Extraction	Extract real-time data collected from the survey, downloadable in Microsoft Excel format

Once the survey is created, the e-mail list can be uploaded and the body of the recipient e-mail can be defined. The user is then prompted to create the waves, or set automatic distribution dates, for the survey to be sent. These waves are automatically sent on the dates defined by the PI, without any additional intervention required.

**Figure 3:**
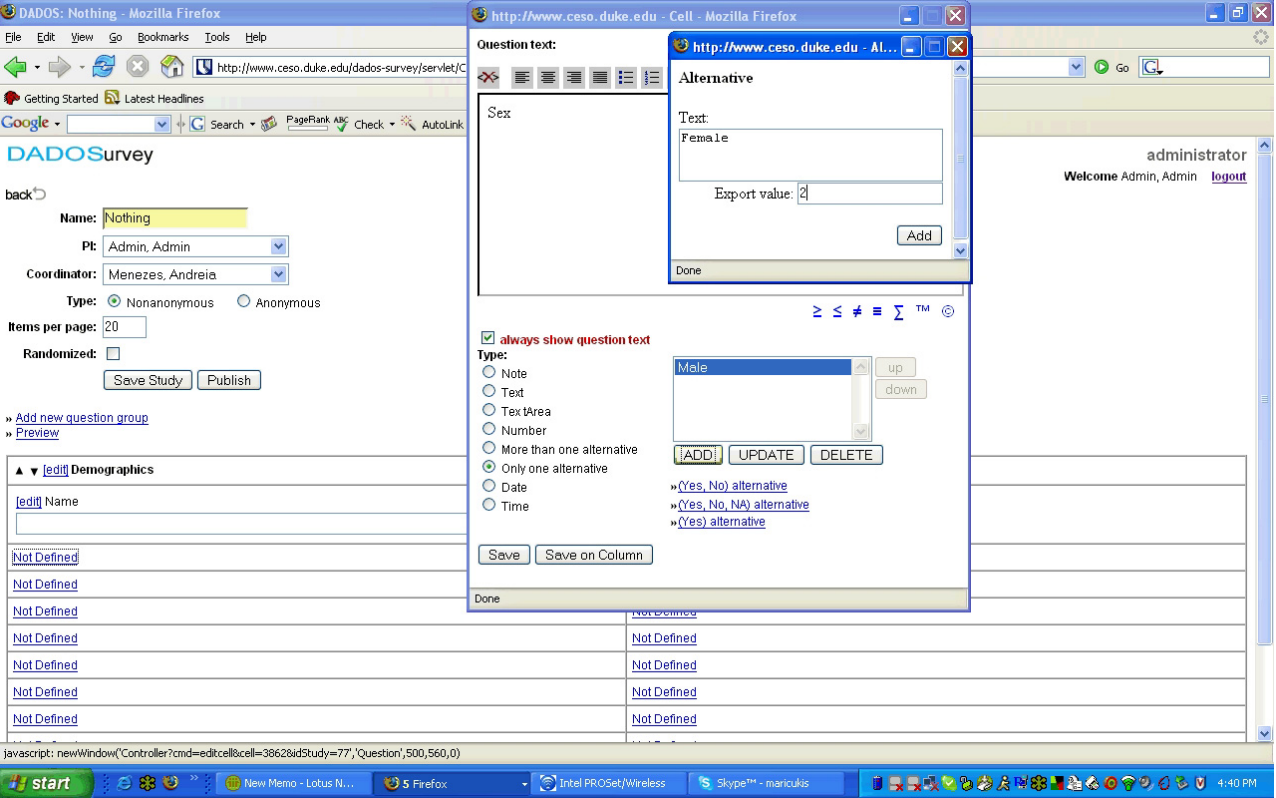
Selection of type of question and optional features.

Each survey question is entered into a text box with capability of adding web links or links to files associated with the question, including most image formats and flash video. The investigator is able to select the type of question and optional features [see Figure 3 and Table 3].

**Table 3:** Explanation of Options for Questions in Survey

Note	Instructions or label for a group of questions
Text	Free-text response that requires response of one line or less
Text Area	Free-text response that requires more than one line
Number	Free-text numeric response
More than one alternative	Response that can include more than once selection of user-defined alternative
Only one alternative	Response that is limited to only one user-defined alternative
Date	Response that is limited to date format
Time	Response that is limited to time format

As Web-survey participants generate data, it is stored on a password-protected, encrypted server at our institution. At any time during the Web-survey study, the PI is able to extract real-time response data that is compiled in a downloadable Microsoft Excel (Microsoft Corporation, Redmond, WA) spreadsheet.

## Results

### Usability

To date, DADOS-Survey has been successfully implemented infive IRB-approved studies conducted by various departments within the Duke University Health System. Each of these studies employed a Web-survey design as their primary methodology. Our initial experience indicates that DADOS-Survey has been used with relative ease by each of the investigators

### Formal usability

Usability testing was performed in accordance with previous novel software applications from our institution [9]. As part of formal usability testing, ten physician-researchers were randomly selected from a departmental directory from the authors' two institutions. Prior to using the application for the first time and subsequently completing the usability survey, each investigator was given a 20-minute, live tutorial by one of the authors (AS) that described the application and all of its functions. These volunteers were first asked to use the program to design a survey and provide their anonymous feedback regarding their experience. In the next phase, these same ten researchers were the recipients of a non-anonymous Web-survey designed and launched using DADOS-Survey. This survey required the researcher to read and agree to an informed consent page. None of the researchers reported any issues with login, informed consent, or answering the brief survey.

The Web-survey questions and their responses are listed in Table 4. The objective of this survey was to evaluate users' overall impressions of DADOS-Survey, from both the perspective of a survey designer and a survey recipient. Formal usability results demonstrated that, from the perspective of researchers, DADOS-Survey presented excellent speed (10/10 users strongly agreed), was extremely easy to learn (6/10 users strongly agreed) and use (9/10 users strongly agreed), had a functionality that was easily understandable (7/10 users strongly agreed), and a navigation that was highly intuitive (7/10 users strongly agreed). No additional comments were provided by the users, nor were any additional features requested during the formal usability analysis phase by any of the testers.

**Table 4:** Formal Usability Testing: Survey Questions and Responses

DADOS-Survey speed is excellent.	Strongly disagree	0/10
	Disagree	0/10
	Neutral	0/10
	Agree	0/10
	Strongly agree	10/10
		
DADOS-Survey is extremely easy to learn	Strongly disagree	0/10
	Disagree	0/10
	Neutral	1/10
	Agree	3/10
	Strongly agree	6/10
		
DADOS-Survey is extremely easy to use	Strongly disagree	0/10
	Disagree	0/10
	Neutral	0/10
	Agree	1/10
	Strongly agree	9/10
		
It is very easy to understand all functionality available within DADOS-Survey (e.g., download files, upload files, etc)	Strongly disagree	0/10
	Disagree	0/10
	Neutral	0/10
	Agree	3/10
	Strongly agree	7/10
		
The navigation in DADOS-Survey is highly intuitive	Strongly disagree	0/10
	Disagree	0/10
	Neutral	1/10
	Agree	2/10
	Strongly agree	7/10

### 2. Field usability

The first four months of field usability were intended to identify and correct minor errors in the software source code, including navigational difficulties, non-functional URL address links, and the improvement of a consistent visual interface throughout the program. This phase of usability testing focused primarily on soliciting feedback and comments from research investigators from the first two research studies that utilized DADOS-Survey. This process allowed for early improvements, as research investigators brought to attention operational issues that could be addressed by software programmers. In doing so, both survey designers and recipients were asked to provide written feedback to one of the authors (MM). Specific feedback from users indicates that DADOS-Survey is "intuitively created" with "no problems" reported in completing the Web-survey design. Two investigators requested that "image capabilities be expanded to include MPEG video file formats."

### Compliance with CHERRIES guidelines for Web-surveys

As noted earlier, DADOS-Survey was designed to ensure total compliance with the CHERRIES guidelines for Web-surveys. Table 5 summarizes the CHERRIES guidelines and lists features of DADOS-Survey that adhere to the CHERRIES recommendations.

**Table 5:** Detailed summary of DADOS-Survey compliance with CHERRIES

**CHERRIES Item Category**	**CHERRIES Checklist Category**	**DADOS-Survey compliance**
Design	Describe survey design	√ Described in the recipient e-mail invitation
IRB approval and informed consent process	IRB approval	√ Information provided in the recipient e-mail invitation and first page of survey
	Informed consent	
	Data protection	
Development and pre-testing	Development and testing	√ Described in the recipient e-mail invitation
Recruitment process and description of the sample having access to the questionnaire	Open survey versus closed survey	√ User selectable option based on IRB approval from host institution
	Contact mode	√ Investigator must report
	Advertising the survey	
Survey administration	Web/E-mail	√ Survey can be posted on the Web or sent over e-mail. Responses are automatically captured by DADOS
	Context	√ Investigator must report
	Mandatory/voluntary	
	Incentives	
	Time/Date	√ Investigator must report. Time/Date data is automatically captured by DADOS.
	Randomization of items or questionnaires	√ DADOS is user configured to randomize/alternate items
	Adaptive questioning	√ DADOS is user configured to conditionally display items
	Number of items	√ DADOS automatically captures and reports this data
	Number of screens (pages)	
	Completeness check	√ DADOS allows user to configure a completeness check based on Java Script. A non-response option is provided to participants. One response and multiple response options are also available.
	Review step	√ Review step allows participants to alter their responses before submission
Response rates	Unique site visitor	√ DADOS captures IP address to determine unique site visitors
	View rate (Ratio unique site visitors/unique survey visitors)	√DADOS computes this information and provides user with the results
	Participation rate (Ratio unique survey page visitors/agreed to participate)	
	Completion rate (Ratio agreed to participate/finished survey)	
Preventing multiple entries from the same individual	Cookies used	For non-anonymous surveys, a user can only answer a survey once. For anonymous surveys, if the user does not complete the survey at once, the data collected is not saved.
	IP check	This was decided not to be implemented so that different users could use the same computer to answer the survey
	Log file analysis	-
	Registration	The registration is done by importing a spreadsheet with the subjects. Once a subject completes the survey he/she cannot log in using the same user again.
Analysis	Handling of incomplete questionnaires	All surveys are analyzed, both completed and uncompleted; uncompleted questions are understood as missing.
	Questionnaires submitted with an atypical timestamp	-
	Statistical correction	-

### Current utilization

DADOS-Survey has been successfully implemented at our institution in a number of studies. Orthopedic specialists have used the software application in Web-surveys of surgeons to assess national and international practice variation in orthopedic surgical procedures. Several Web-surveys examining patient satisfaction are underway, both with and without private health information contained within the survey. Lastly, faculty members in the Department of Radiology at our institution have used DADOS-Survey to design inter- and intra-observer agreement studies using high-resolution clinical images.

## Discussion

We have demonstrated that DADOS-Survey has the potential to have an important role in the future direction of Web-survey administration in biomedical research. This CHERRIES-compliant application is tailored to the emerging requirements of quality data collection in medicine. Of note, the CONSORT guidelines have had a drastic impact on the design, implementation, and reporting of randomized trials [10-12]. Similarly, the authors feel that compliance with CHERRIES requires that its guidelines be implemented at the design stage of a Web-survey. DADOS-Survey allows for such compliance in its software design. By allowing the user to create and distribute CHERRIES-compliant surveys in minimal time, it is expected that the free distribution of DADOS-Survey will make it an efficacious and attractive alternative to traditional paper surveys for researchers worldwide to conduct studies compliant with the recently developed CHERRIES data reporting guidelines.

DADOS-Survey has several advantages over existing Web-based survey applications (Survey Solutions Express, Perseus Development Corporation, Braintree, MA; Inquisite, Austin, TX; Vista, Vanguard Software Corporation, Cary, NC). To the best of our knowledge, this is the first freely distributed open-source application that incorporates compliance with the CHERRIES data reporting guidelines into its design. In contrast to the commercial packages, which do not permit modification of the source code, we are confident that the modifiable source code of DADOS-Survey will encourage users to customize and further improve the features in this release. Importantly, DADOS-Survey can be installed on any academic institutional server, allowing users to securely store and manage data at their own institutions. One disadvantage of DADOS-Survey over commercial survey applications is that users will likely need to consult their institutional Information Technology division for assistance in installation and in modifying the source code.

### Potential uses

1. DADOS-Survey allows researchers to develop randomized experiments on the Web in which different questions are assigned to different subjects. An example includes the presentation of statements about drug efficacy while randomly choosing whether or not to introduce a picture of a visual complication, with the goal of evaluating whether psychological factors play a role in the evaluation of medical evidence.

2. In contrast to most commercial Web-based survey applications, the source code for DADOS-Survey is user customizable and can be installed on an academic institutional server, allowing data to be collected in a manner consistent with institution-specific and HIPAA data privacy regulations. Patient data can be collected with a greater degree of confidence among patients regarding the security of their responses. This includes surveys in which patients are asked about their perception of risk factors for certain medical conditions or their ethical perceptions.

3. Inter and intra-observer reliability studies evaluating radiographic imaging with dynamic images, including fluoroscopy cine images. The freely modifiable source code allows institutional users to develop plug-ins for DADOS-Survey to utilize different formats of images and video in surveys. Although movies and Java-script can be incorporated in the current version at our own institution, such features require programming that is not accessible to general users at the present time. Future versions of DADOS-Survey should include the ability by general users to dynamically insert movies and Java-script code into their survey designs.

## Conclusion

We believe that DADOS-Survey is the first freely distributed open-source Web-survey software application that facilitates compliance with the CHERRIES data reporting guidelines. The initial experiences among researchers (i.e. survey designers) and those of survey recipients in early research studies have been positive. It is hoped that additional features will continue to be added to DADOS-Survey to increase its functionality and customization for unique Web-survey methodologies.

## Availability and requirements

Project Name: DADOS-Survey

Project Home Page:  (click link for "Free software")

Operating System: Linux/Windows

Programming Language: Java >= 1.4.2

Other requirements: Tomcat 5.x, MySQL 5.0

License: GNU General Public License

Any restrictions to use by non-academics: none

## List of abbreviations used

CHERRIES – Checklist for Reporting Results of Internet E-Surveys

HIPAA – Health Insurance Portability and Accountability Act

PHI – Protected Health Information

## Competing interests

The author(s) declare that they have no competing interests.

## Authors' contributions

All authors read and approved the final manuscript.

AS – designed the application, assisted with software testing, conducted the usability tests, and wrote the first draft of the manuscript

DOJ – assisted with the design of the application, reviewed the manuscript for intellectual content

HM – assisted with the design of the application, wrote and tested the source code for the application, reviewed the manuscript for intellectual content

MH – assisted with the design of the application, reviewed the manuscript for intellectual content

AM – assisted with the design of the application, assisted with field-testing, reviewed the manuscript for intellectual content

MM – assisted with the design of the application, assisted with field-testing and software debugging, reviewed the manuscript for intellectual content

RP – designed the application, assisted with software testing, conducted the usability tests, and wrote the first draft of the manuscript

## Pre-publication history

The pre-publication history for this paper can be accessed here:


